# Supplementary figures and images for: Function of Cajal Bodies in Nuclear RNA Retention in A. thaliana Leaves Subjected to Hypoxia
Source: Int J Mol Sci. 2022 Jul 8;23(14):7568. doi: 10.3390/ijms23147568 (PMC9321658; doi:10.3390/ijms23147568)

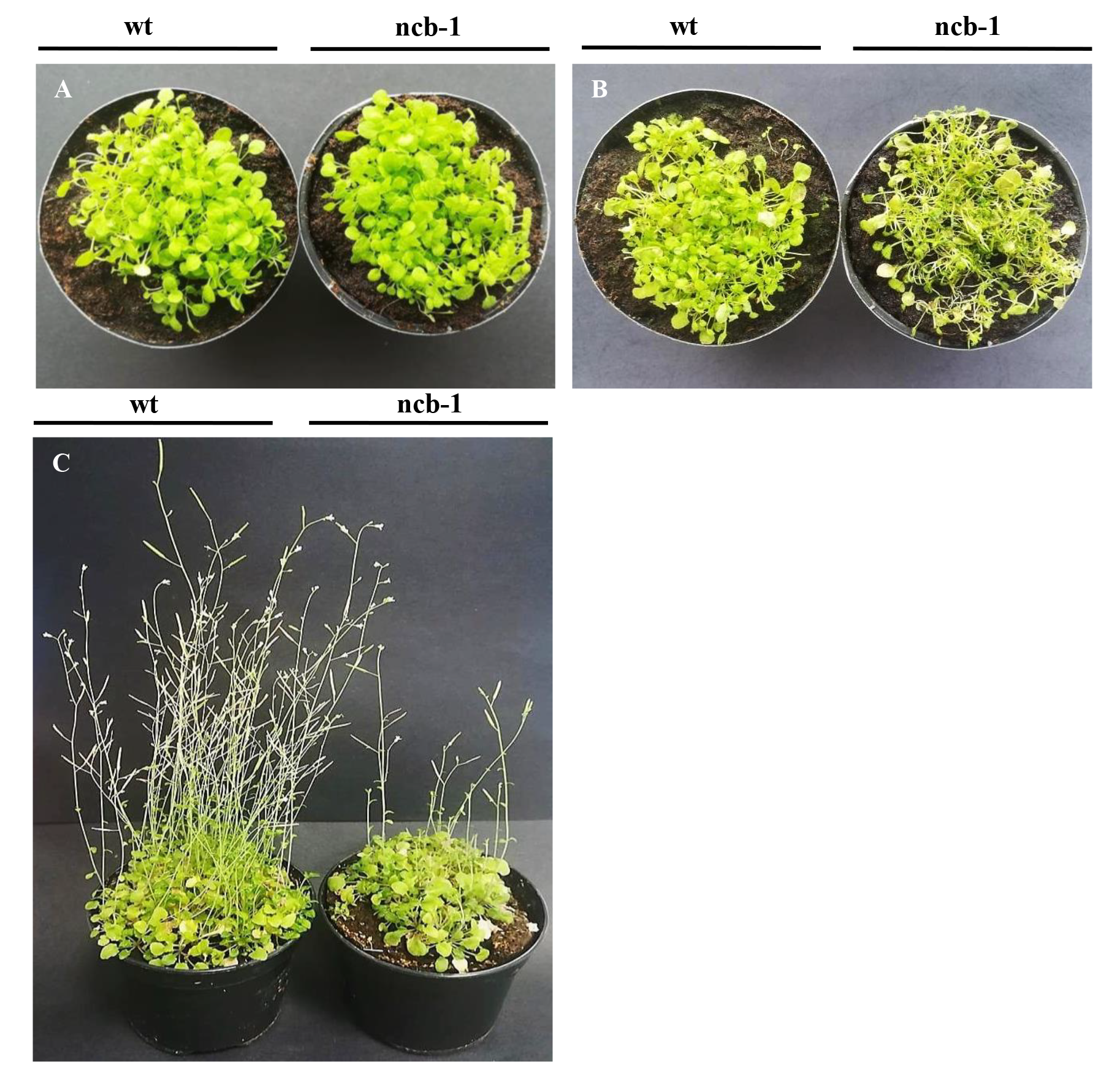

Supplement: Supplementary file 1 [file ijms-23-07568-s001.zip › Figure S1.tif]

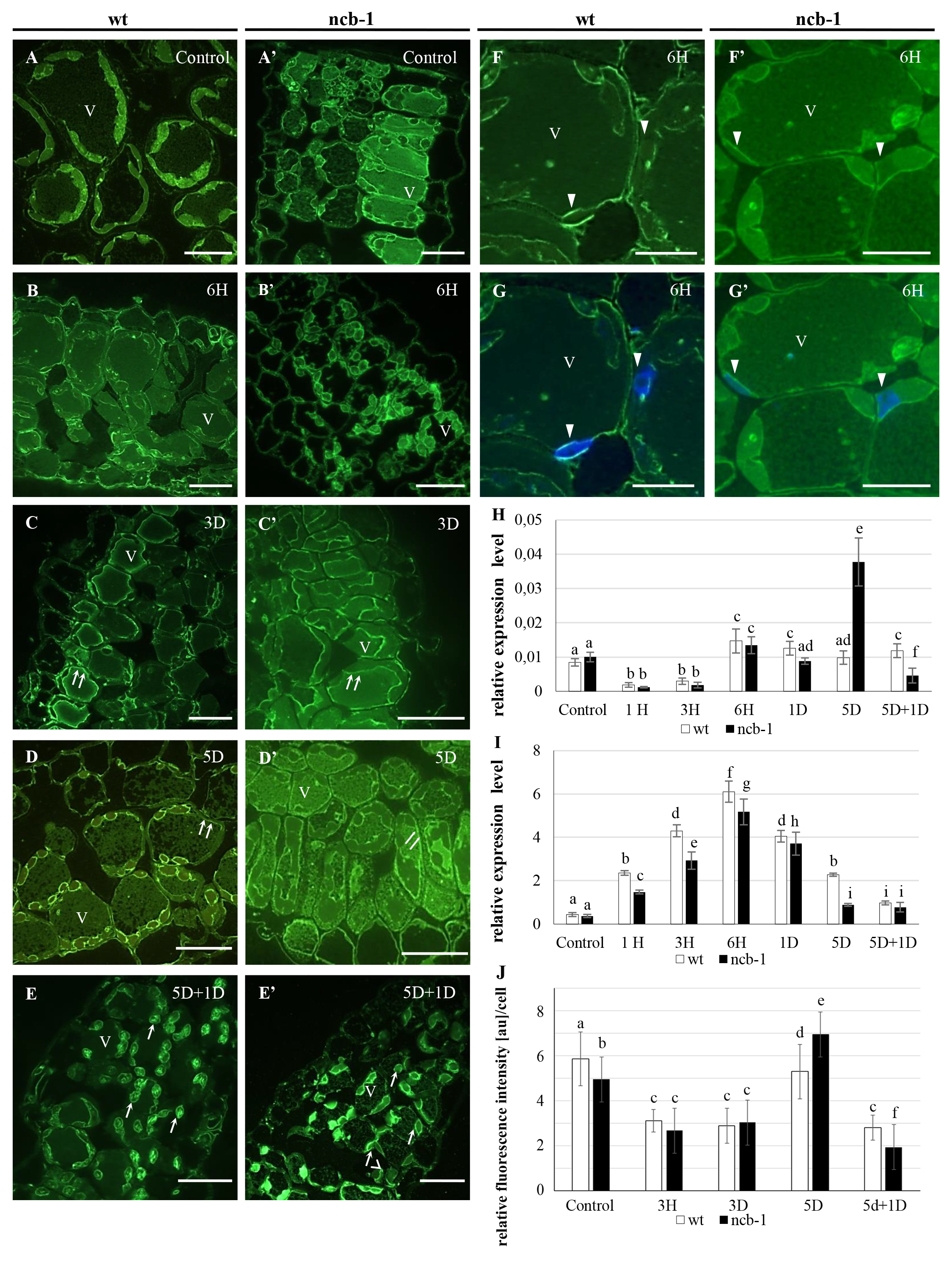

Supplement: Supplementary file 1 [file ijms-23-07568-s001.zip › Figure S2.tif]

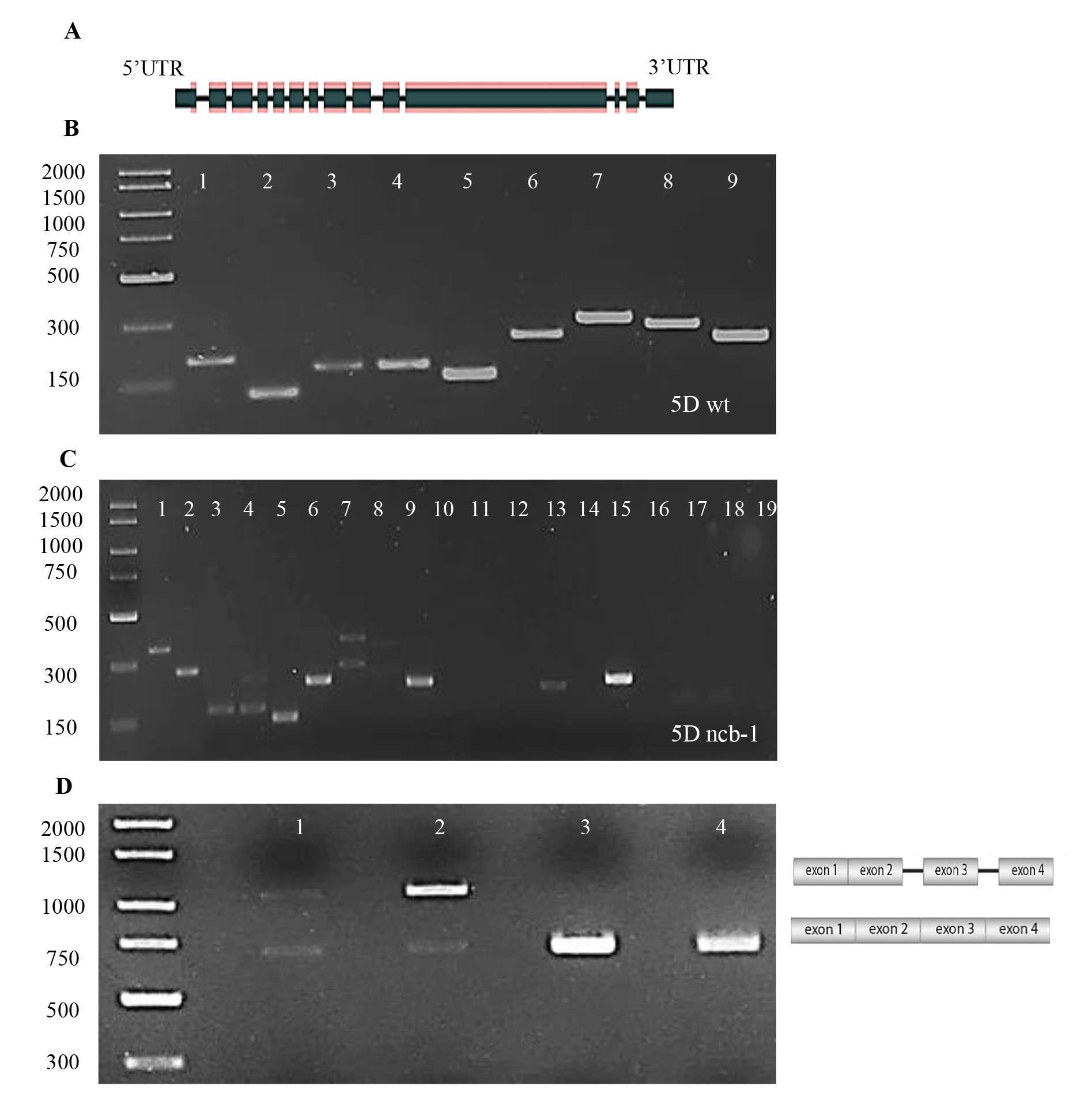

Supplement: Supplementary file 1 [file ijms-23-07568-s001.zip › Figure S3.tif]

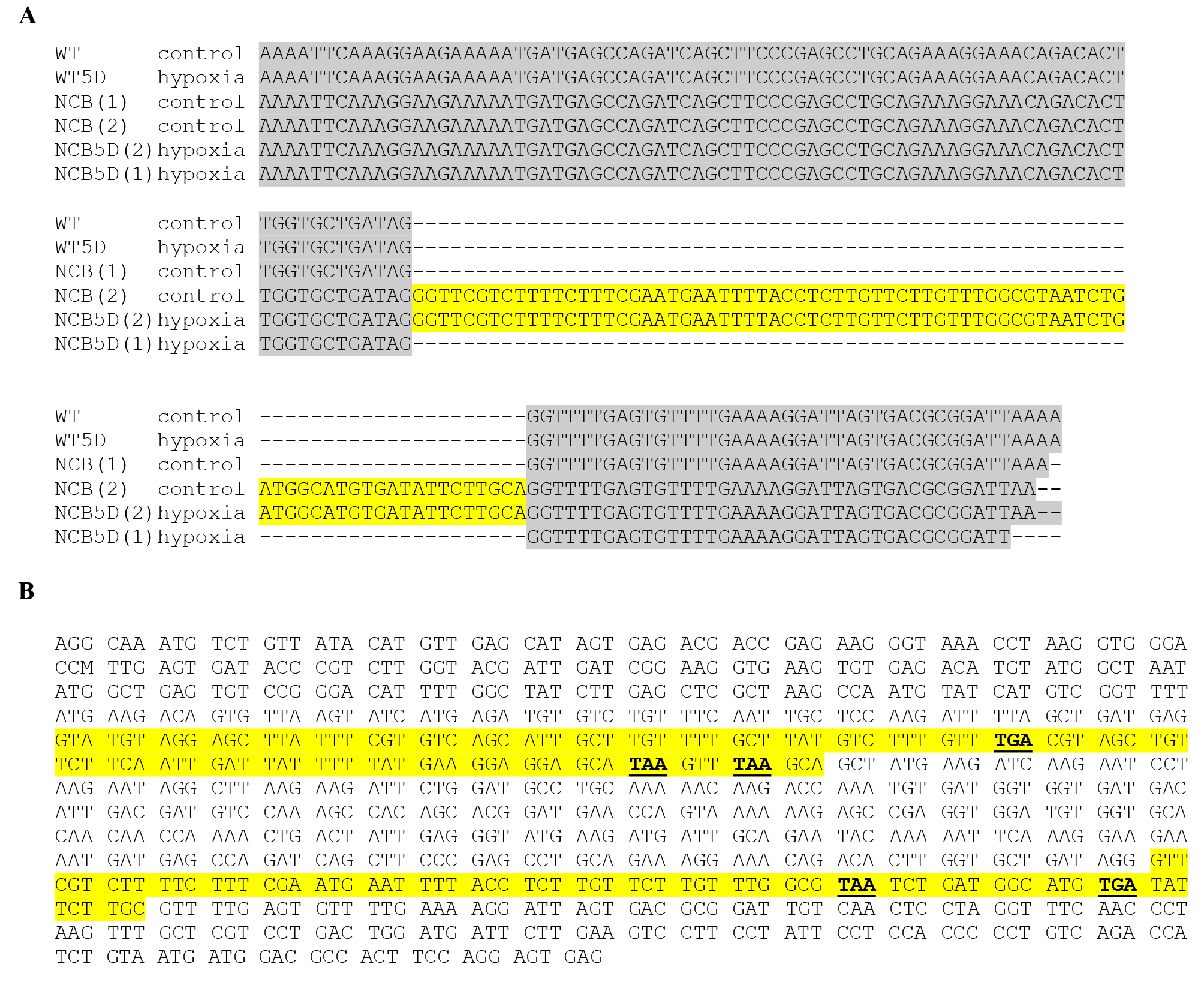

Supplement: Supplementary file 1 [file ijms-23-07568-s001.zip › Figure S4.tif]

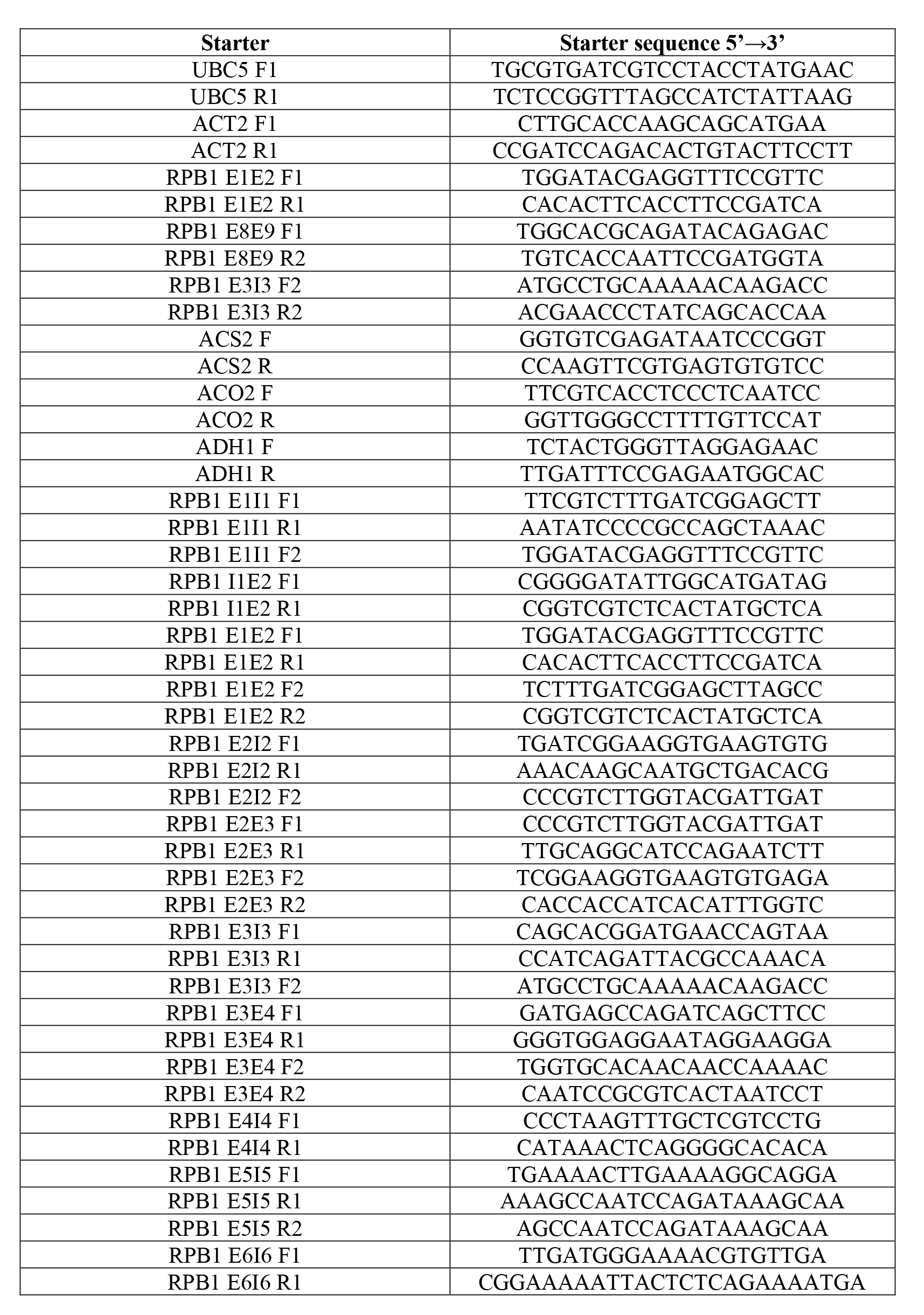

Supplement: Supplementary file 1 [file ijms-23-07568-s001.zip › Figure S5.tif]
